# Are Variants Causing Cardiac Arrhythmia Risk Factors in Sudden Unexpected Death in Epilepsy?

**DOI:** 10.3389/fneur.2020.00925

**Published:** 2020-09-08

**Authors:** Lauren E. Bleakley, Ming S. Soh, Richard D. Bagnall, Lynette G. Sadleir, Samuel Gooley, Christopher Semsarian, Ingrid E. Scheffer, Samuel F. Berkovic, Christopher A. Reid

**Affiliations:** ^1^Florey Institute of Neuroscience and Mental Health, University of Melbourne, Parkville, VIC, Australia; ^2^Agnes Ginges Centre for Molecular Cardiology Centenary Institute, The University of Sydney, Sydney, NSW, Australia; ^3^Faculty of Medicine and Health, University of Sydney, Sydney, NSW, Australia; ^4^Department of Paediatrics and Child Health, University of Otago, Wellington, New Zealand; ^5^Department of Medicine, Epilepsy Research Centre, Austin Health, University of Melbourne, Heidelberg, VIC, Australia; ^6^Department of Paediatrics, Royal Children's Hospital, University of Melbourne, Melbourne, VIC, Australia

**Keywords:** sudden unexpected death in epilepsy, epilepsy, cardiac arrhythmia, genetics, ion channels, common variants

## Abstract

Sudden unexpected death in epilepsy (SUDEP) is the most common cause of premature mortality in individuals with epilepsy. Acute and adaptive changes in heart rhythm in epilepsy implicate cardiac dysfunction as a potential pathogenic mechanism in SUDEP. Furthermore, variants in genes associated with Long QT syndrome (LQTS) have been identified in patients with SUDEP. LQTS is a cardiac arrhythmia condition that causes sudden cardiac death with strong similarities to SUDEP. Here, we discuss the possibility of an additive risk of death due to the functional consequences of a pathogenic variant in an LQTS gene interacting with seizure-mediated changes in cardiac function. Extending this general concept, we propose a hypothesis that common variants in LQTS genes, which cause a subtle impact on channel function and would not normally be considered risk factors for cardiac disease, may increase the risk of sudden death when combined with epilepsy. A greater understanding of the interaction between epilepsy, cardiac arrhythmia, and SUDEP will inform our understanding of SUDEP risk and subsequent potential prophylactic treatment.

## Introduction

People with epilepsy have a two- to threefold increased risk of premature mortality, with sudden unexpected death in epilepsy (SUDEP) the most common epilepsy-related cause (1–5). SUDEP is defined as “a sudden, unexpected, witnessed or unwitnessed, non-traumatic and non-drowning death, occurring in benign circumstances, in an individual with epilepsy, with or without evidence for a seizure and excluding documented status epilepticus, in which postmortem examination does not reveal a cause of death” (6). The incidence of SUDEP varies widely depending upon the subpopulation of interest, from 0.2 per 1,000 persons per year in children, and 1.2 per 1,000 persons per year in adults (7), to 2.46 or higher per 1,000 persons per year in people with refractory epilepsy (8, 9), and up to 10 per 1,000 persons per year in candidates for epilepsy surgery (10). In addition, certain severe epilepsy syndromes, such as the Developmental and Epileptic Encephalopathies (DEEs), place individuals at a greater risk of sudden death (11). A well-established example of this is Dravet syndrome, an intractable infantile-onset DEE mainly caused by pathogenic variants in *SCN1A* (12, 13). Dravet syndrome has a mortality rate of 16 per 1,000 persons per year, which translates to a 17% mortality risk in the first two decades of life with 59% of these deaths due to SUDEP (14). As expected, many rare variants in other genes that are closely associated with DEE have been identified in studies exploring the genetic architecture of SUDEP (11, 15).

Risk factors have been identified for SUDEP, with the most important being that the individual experiences tonic–clonic seizures, especially if they occur with high frequency (11, 16, 17). Other risk factors include epilepsy duration, age of onset of epilepsy, frequent changes in antiseizure medication doses, and, in some studies, antiepileptic drug polytherapy (3, 11, 16, 18). However, SUDEP can also occur in individuals with mild types of epilepsy (19, 20), as well as those with well-controlled epilepsy (3, 20), suggesting that other risk factors exist.

The pathophysiological mechanism(s) responsible for SUDEP remain largely unclear, despite considerable interest and research endeavor. A prevailing hypothesis is that SUDEP occurs following centrally mediated autonomic failure, which is most likely triggered by a tonic–clonic seizure (21). This hypothesis is strongly supported by a seminal paper, which reported findings from a systematic retrospective survey of SUDEP deaths that occurred in epilepsy video-electroencephalogram (video-EEG) monitoring units. This study found that SUDEP followed a consistent pattern whereby individuals had a tonic–clonic seizure (most were focal to bilateral tonic–clonic, and some were generalized tonic–clonic), followed by a period of rapid breathing, and then cardiorespiratory dysfunction leading to terminal apnea and asystole (22). Of particular note is the finding that terminal apnea always preceded terminal asystole (22). However, while these results are compelling, they are unlikely to be telling the whole story. The patient population in this study was small and involved individuals who were undergoing long-term monitoring, implying refractory epilepsy—a selected subset of patients who we know are at greater risk of SUDEP. Such a subset does not provide a representative sample of all individuals with SUDEP, raising the possibility that other pathological mechanisms can also cause SUDEP. One such mechanism, which has been widely studied, is the presence of abnormal cardiac rhythms (4, 15).

**Figure 1:**
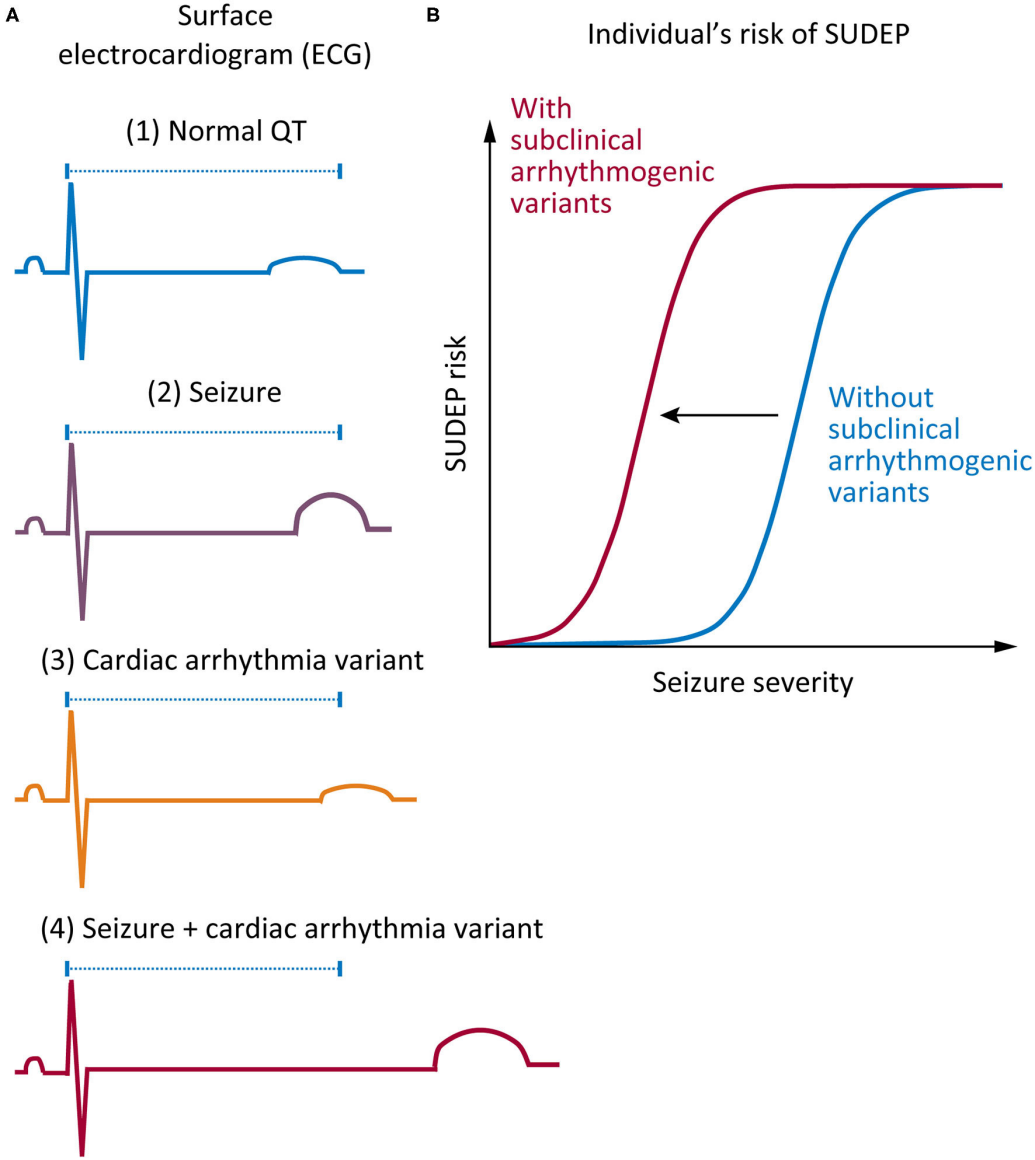
Proposed mechanism on how genetic variants associated with cardiac arrhythmia may increase risk of sudden unexpected death in epilepsy (SUDEP). **(A)** Comparison of surface electrocardiogram (ECG) in various conditions. (1) A normal QT interval in a healthy individual without epilepsy or cardiac arrhythmia (blue). (2) A small shift in the QT interval might occur in a patient with epilepsy during a seizure (purple). (3) A “subclinical” individual carrying a functional variant in a cardiac arrhythmia gene, which only subtly shifts the QT interval (orange). (4) A combination of a “subclinical” variant and a seizure event results in a nonlinear increase in the QT interval, thus increasing the risk of sudden death (red). **(B)** The increased risk of sudden death that is conferred by harboring a “subclinical” functional variant in a cardiac gene presents itself in the acute context of a seizure, which causes disruption of normal cardiac function. Such cardiac disruption is more likely with longer seizures, seizures with more intense autonomic discharges, and perhaps more violent seizures with more systemic metabolic changes, all effects encapsulated by the term “seizure severity.” This risk is present on every occasion when an affected individual has a seizure.

There are several lines of evidence supporting a role for cardiac arrhythmia in SUDEP. First, there are clear similarities between SUDEP and sudden cardiac death: in both cases, death is unexpected, and no cause of death is identified after comprehensive postmortem (23). Second, both human and animal studies show that seizure-mediated changes in cardiac electrophysiology occur, including seizure-driven cortical autonomic dysfunction and altered cardiac ion channel expression (24). Finally, recent genetic studies have found variants in genes associated with cardiac arrhythmia syndromes in some individuals with SUDEP (15, 25–28). Here, we propose that there may be an interaction between seizure activity and increased risk of sudden death in epilepsy patients who harbor variants (including common variants) in arrhythmogenic genes (Figure 1).

## Arrhythmogenic Genes in SUDEP

A number of studies have explored the genetic architecture of SUDEP (25, 26, 29, 30). These studies have identified variation in several genes known to cause cardiac arrhythmia syndromes—in particular, long QT syndrome (LQTS)—and sudden death. LQTS is a cardiac arrhythmia syndrome resulting from delayed myocardial repolarization that manifests as a prolonged QT interval on the electrocardiogram (ECG) (31). This increases the risk of “torsades de pointes,” a distinctive form of ventricular tachycardia (32, 33) that can trigger sudden cardiac death in otherwise healthy individuals with structurally normal hearts (31). About 75% of familial LQTS is accounted for by three major genes, *KCNQ1, KCNH2*, and *SCN5A* (31). Variants in these three genes (25–28), and in several other cardiac and LQTS genes (25, 34), have been identified in SUDEP cases (Table 1).

**Table 1:** Nonsynonymous variants in cardiac arrhythmia genes that have been identified in sudden unexpected death in epilepsy (SUDEP).

**Gene**	**Variant**	**gnomAD allele count**	**Sorting intolerant from tolerant (SIFT)**	**PolyPhen-2**	**References**
*AKAP9*	Ile1749Thr	195	Deleterious	Probably damaging	(25)
	Arg2607Gly	0	Deleterious	Probably damaging	(25)
*ANK2*	Ala1027Asp	0	Deleterious	Probably damaging	(25)
	Ser2440Asn	0	Deleterious	Probably damaging	(25)
	Ile3903Asn	1	Deleterious	Probably damaging	(25)
*HCN1*	Gly46Val	0	Deleterious	Benign	(34)
*HCN2*	Phe738Cys	0	Tolerated	Probably damaging	(34)
	Pro802Ser	10	Tolerated low confidence	Benign	(34)
*HCN3*	Lys69Arg	3,014	Tolerated	Benign	(34)
	Pro630Leu	6,780	Deleterious low confidence	Benign	(34)
*HCN4*	Gly36Glu	7,166	Deleterious low confidence	Benign	(34)
	Val759Ile	870	Tolerated	Benign	(34)
	Gly973Arg	19	Tolerated low confidence	Possibly damaging	(34)
	Arg1044Trp	4	Deleterious low confidence	Probably damaging	(34)
	Glu1193Gln	205	Tolerated low confidence	Probably damaging	(25)
*KCNH2*	Ile82Thr	0	Deleterious	Benign	(28)
	Arg176Trp	44	Deleterious	Possibly damaging	(26)
	Arg744^*^	0			(25)
	Gly749Ala	0	Deleterious	Possibly damaging	(25)
	Gly924Ala	8	Tolerated	Possibly damaging	(25)
	Arg1047Leu	3,117	Tolerated	Possibly damaging	(26)
*KCNQ1*	Tyr662*	12			(25)
*RYR2*	Cys1489Arg	40	Tolerated	Benign	(25)
*SCN5A*	Val223Gly	0	Deleterious	Probably damaging	(25)
	Ile397Val	1	Deleterious	Probably damaging	(25)
	Arg523Cys	2	Deleterious	Benign	(27)
	His558Arg	62,556	Tolerated	Benign	(26)
	Ala572Asp	1,451	Tolerated	Benign	(26)
	Pro1090Leu	458	Tolerated	Benign	(26)
	Pro2006Ala	252	Tolerated low confidence	Benign	(26)

* *indicates a nonsense mutation resulting in a premature stop codon*.

It is important to highlight that the presence alone of a rare variant in a case of SUDEP does not confirm its pathogenicity or contribution to SUDEP risk. Furthermore, in cases of sudden death, where seizures have been diagnosed, but where pathogenic variants in cardiac genes are present, other potential explanations—such as misdiagnosis of convulsive syncope due to a cardiac cause, a common occurrence in individuals with LQTS—must be ruled out. This does not always occur (35, 36). A recent systematic review has highlighted the issues in diagnosis of SUDEP and the challenges in inferring causation in cases with variants in cardiac genes (37). That being said, current genetic findings that associate variants in cardiac arrhythmia genes with SUDEP do allow for some discussion and hypothesis generation about pathogenic mechanisms.

## Potential Impact of Seizures in Patients Harboring Pathogenic Variants in Arrhythmogenic Genes

The LQTS pathogenic variants in *KCNH2*, p.Arg744^*^, and p.Gly924Ala, have each been identified in a SUDEP patient (25). It is intuitive to think that an individual with epilepsy who also harbors a pathogenic cardiac genetic variant will be at higher risk of sudden unexpected death. The finding of validated LQTS variants in SUDEP provides indirect evidence supporting this premise (Table 1). It is also well-recognized that numerous acute postictal arrhythmia patterns occur in epilepsy patients, presumably through significant changes in autonomic function (38). It is possible that these rhythm changes could interact with the physiological consequences of harboring arrhythmogenic variants to increase the risk of sudden death (Figure 1). Data from the Kcnq1 p.Thr311Ile mouse model of LQTS provides more direct evidence that an interaction between acute seizures and elongation of the QT interval may occur. This mouse has a prolonged QT interval and frequent seizures relative to controls (39, 40), and interestingly, over half of ECG-detected cardiac abnormalities are associated with epileptiform discharges on the EEG (40). Furthermore, we know that in many epilepsy patient populations and animal models of epilepsy, basal ECG properties are changed (24). In patients who also harbor underlying variants in arrhythmogenic genes, these long-term changes in heart rhythm may increase the risk of sudden death. It will be challenging to rigorously test whether seizures increase the risk of sudden death in patients harboring pathogenic LQTS variants. Designs could include cross-sectional analyses of families with LQTS and determining if the death rate is higher in those with known coexistent epilepsy or longitudinal follow-up of subjects with epilepsy stratified into those with and without known LQTS variants.

## Can “Subclinical” Variants in Arrhythmogenic Genes Contribute to SUDEP Risk?

Consider a potentially more broadly applicable situation, where a patient with epilepsy harbors a common variant in a gene associated with cardiac arrhythmia, but the variant is not normally associated with clinical events. Such a “subclinical” variant alters cardiac function but to a degree that is below the threshold to cause clinically recognized LQTS; thus, it would not normally be considered a risk factor for cardiac disease. Could patients such as this be at an increased risk of death during or immediately following seizures (Figure 1)? We propose that small shifts in the QT interval, which are present in seizures and independently in people harboring common variants in arrhythmogenic genes that cause minor changes in channel function, will only increase the risk of sudden death slightly. However, when combined, seizures and a common variant in an arrhythmogenic gene may interact to significantly increase SUDEP risk.

There is a precedent for this idea in a recently published study that investigated the relationship between *KCNQ1* common variants and sudden death during illegal drug use. The missense variant KCNQ1 p.Gly643Ser (found 1,433 times in the gnomAD database) was more common in patients who died of drug-related causes than in the general population (41). Interestingly, this common variant has been found to cause a mild loss-of-function of the K_v_7.1 voltage-gated potassium channel when studied in a heterologous expression assay (42, 43). This finding provides evidence that a common variant, which is unlikely to be pathogenic in isolation, could potentially increase the risk of death under certain circumstances.

Numerous common variants in LQTS genes have been identified in SUDEP patients (25–28) (Table 1). This is exemplified by variation in *KCNH2*, a cardiac gene that encodes the α subunit of the voltage-gated potassium channel K_v_11.1 (44). The common KCNH2 p.Arg1047Leu variant (found >3,000 times in gnomAD) has been observed in four patients that have suffered SUDEP (26). Functional testing of this variant suggests that it causes mild loss of channel function (45). It is well-established that loss-of-function *KCNH2* variants cause LQTS type 2, which does predispose individuals to greater risk of sudden death (31, 44, 46, 47). As such, although the KCNH2 p.Arg1047Leu variant is not disease causing in its own right, the mild loss-of-function that it causes positions it as a potential risk factor for SUDEP. Owing to the prevalence of this variant in the population, very large sample sizes would be needed to show a statistical association with SUDEP, with a similar issue arising with all common variants in LQTS genes that are identified in patients.

## Conclusion and Potential Clinical Implications

For patients with epilepsy and their families, SUDEP is a frightening possibility, made even more so by its unpredictability. SUDEP is undoubtedly a highly heterogeneous condition, and a given individual's risk is likely to be determined by a complex interaction of many contributing factors. These include both genetic and environmental risk factors for mechanisms as diverse as seizures, cardiac arrhythmias, respiratory dysfunction, and autonomic dysfunction (15). Reducing seizure frequency and severity is important in reducing SUDEP risk. However, in such a multifactorial condition, the potential risk conferred by other mechanisms—such as cardiac arrhythmias—also warrants consideration. Variants in diverse cardiac arrhythmia genes have been found in SUDEP patients (25–29, 34), but we clearly need a much better understanding of the impact of these variants in the context of an individual with epilepsy.

Although it seems obvious, an increased risk of death associated with an epilepsy patient having a validated LQTS variant has not been shown. It is also not known whether SUDEP risk increases in cases of epilepsy when a patient harbors one or more variants of unknown significance in cardiac arrhythmia genes, such as in the case of the patients with the KCNH2 p.Arg1047Leu common variant outlined above. We propose that genetic studies in SUDEP should be extended to include the characterization of common variants in cardiac arrhythmia genes. Additionally, functional studies are required to identify if these variants cause subclinical biophysical changes, which could exacerbate the risk of sudden death during a seizure. Currently, the prevalence of rare variants with functional impact is unknown. Without such information, and indeed until such time as the genetic architecture of SUDEP overall is understood, we are unable to predict the relative contribution of genetic cardiac dysfunction to SUDEP incidence and risk.

Identifying and confirming genetic risk factors that predispose patients with epilepsy to cardiac arrhythmia would have therapeutic implications. Patients identified to be at risk of cardiac arrhythmia could be advised to avoid medications that interact with cardiac ion channels; commence prophylactic treatment with beta blockers, which are used effectively in LQTS to reduce the risk of life-threatening arrhythmias (48); or even consider the implantation of an internal defibrillator or pacemaker to reduce risk of sudden death (49), although evidence for the benefit of such an approach is currently limited (50). Concurrent EEG and ECG monitoring may also be warranted to gain greater insight into the interactions between seizures and cardiac rhythm.

In summary, here we highlight that variants in cardiac genes, both those known to be pathogenic and also those that are currently thought of as “subclinical,” are potential contributors to SUDEP risk. However, there is currently a lack of direct evidence for cardiac variants increasing the risk of SUDEP, demonstrating a need for further research. Given the sample-size challenges of clinical research in this area, we suggest that exploring this hypothesis in experimental animals is a useful next step.

## Data Availability Statement

All datasets generated for this study are included in the article/supplementary material.

## Author Contributions

LB, MS, RB, LS, CS, IS, SB, and CR developed the concept. LB and CR wrote the manuscript. MS generated the figure. All authors contributed to revising and editing the manuscript and approved the submitted version.

## Conflict of Interest

LS was a consultant for the Epilepsy Consortium, has received travel grants from Sequirus and Nutricia, and has received research funding from Zynerba, the Health Research Council of New Zealand and Cure Kids New Zealand. IS has served on scientific advisory boards for UCB, Eisai, GlaxoSmithKline, BioMarin, Nutricia, Rogcon, and Xenon Pharmaceuticals; has received speaker honoraria from GlaxoSmithKline, UCB, BioMarin, Biocodex and Eisai; has received funding for travel from UCB, Biocodex, GlaxoSmithKline, Biomarin and Eisai; has served as an investigator for Zogenix, Zynerba, Ultragenyx, GW Pharma, UCB, Eisai, Anavex Life Sciences, Ovid Therapeutics, Epigenyx, Encoded Therapeutics and Marinus; and has consulted for Zynerba Pharmaceuticals, Atheneum Partners, Ovid Therapeutics, Epilepsy Consortium and UCB. SB declares unrestricted educational grants from UCB Pharma, SciGen and Eisai and consultancy fees from Praxis Precision Medicines. The remaining authors declare that the research was conducted in the absence of any commercial or financial relationships that could be construed as a potential conflict of interest.

## Abbreviations

DEE: developmental and epileptic encephalopathy
ECG: electrocardiogram
EEG: electroencephalogram
LQTS: long QT syndrome
SUDEP: sudden unexpected death in epilepsy.
